# Ptomaines

**Published:** 1898-07

**Authors:** 


### SELECTIONS.

### Ptomaines.

The following definition of a ptomaine is given in the first chapter of Vaughan and Novy's work on ptomaines and leucomaines:

"A ptomaine is a chemical compound which is basic in its character, and which is formed during the putrefaction of organic matter. The name was suggested by Selmi, and is derived from the Greek word ptoma (cadaver). On account of their basic properties, in which they resemble the vegetable alkaloids, ptomaines may be called putrefactive alkaloids. They have been called animal alkaloids, but this is a misnomer, because some ptomaines are formed by putrefaction of vegetable matter. * * * While some of the ptomaines are highly poisonous, this is not an essential property, for others are wholly inert. Indeed the greater number of those which have been isolated up to the present time are not poisonous. On the other hand, all poisonous substances formed during putrefaction are not ptomaines. Thus phenol and hydrogen sulphide are poisonous products of putrefaction, but are not ptomaines. All ptomaines contain nitrogen as an essential part of their basic character. In this, also, they resemble the vegetable alkaloids. Some of them contain oxygen, while others do not. The latter correspond to the volatile vegetable alkaloids, nicotine and conine, and the former correspond to the fixed alkaloids."

A ptomaine which has proved particularly dangerous to health, and even life, is tyrotoxicon. This occurs in milk and cheese. Unfortunately the conditions under which it forms are not fully understood and its presence is not made known by any change in the taste of the food. Many cases of poisoning by ice cream have been ascribed to the presence of tyrotoxicon.

Several methods have been proposed for the extraction of these bodies, none of which are wholly satisfactory. That known as the Stas-Otto method seems, with some modifications, to most nearly meet the wants of investigators. It depends on the facts that the salts of the ptomaines are soluble in water and alcohol and generally insoluble in ether, and that the free alkaloids are soluble in ether. The substance is extracted by 90 percent alcohol, to which a small proportion of tartaric, oxalic or acetic acid has been added; the clear liquid evaporated; the residue taken up with absolute alcohol; the liquid again evaporated; the residue dissolved in water and shaken with ether. The ether being separated, on evaporation yields the alkaloid.

